# Acute portal vein thrombosis postimage-guided liver biopsy in common variable immunodeficiency

**DOI:** 10.1016/j.radcr.2026.06.039

**Published:** 2026-07-04

**Authors:** Satya K. Morar, Kamal N. Morar

**Affiliations:** a School of Medicine, Case Western Reserve University, Cleveland, OH; b Division of Vascular & Interventional Radiology, University of Cincinnati Health System, Cincinnati, OH

**Keywords:** Portal vein thrombosis (PVT), Percutaneous liver biopsy, Cirrhosis, Common variable immunodeficiency (CVID), Liver biopsy complications, Interventional radiology

## Abstract

Portal vein thrombosis (PVT) is a rare complication following percutaneous liver biopsy, with an incidence below 0.4% in large multicenter series. We report a 51-year-old male with common variable immunodeficiency (CVID), chronic liver enzyme elevation, and preoperative imaging consistent with cirrhotic morphology, who developed acute abdominal pain within minutes of ultrasound-guided liver biopsy. Notably, biopsy demonstrated only mild steatosis with portal chronic inflammation and no significant fibrosis—a discordance from imaging attributed to CVID-associated nodular regenerative hyperplasia and biopsy sampling error. Contrast-enhanced CT on the day of the procedure showed heterogeneous left lobe enhancement consistent with a transient hepatic attenuation difference (THAD) secondary to focal portal venous obstruction, with a subsegmental filling defect in segment 3 on portal venous phase imaging. Repeat CT on postprocedure day 1 demonstrated thrombus extension into the left main portal vein; MRCP excluded biliary complications and hemobilia. The patient was treated with intravenous heparin transitioned to oral apixaban, with symptomatic improvement and normalization of liver function tests. A formal hypercoagulable evaluation was not performed given the provoked clinical context and normal INR, and no postdischarge imaging was available to assess recanalization; both are acknowledged as limitations. This case illustrates the convergence of multiple risk factors—abnormal hepatic vascular architecture from CVID-related liver disease, endothelial injury from needle passage, and potential hemostatic agent migration—in the pathogenesis of postbiopsy PVT, and underscores the importance of prompt vascular imaging and anticoagulation in patients presenting with acute abdominal pain following liver biopsy.

## Introduction

Percutaneous liver biopsy remains a cornerstone in the diagnostic evaluation of hepatic pathology, offering histologic insight into a wide range of liver diseases. While the procedure is generally considered safe, it carries a small but meaningful risk of complications, including hemorrhage, hemobilia, and vascular injury [1,2]. Among these, portal vein thrombosis (PVT) is an infrequent but potentially serious event. A multicenter study of over 68,000 biopsies reported a complication rate below 0.4%, with PVT being exceptionally uncommon [3].

PVT may arise from direct trauma to a portal venous branch during needle passage, local inflammation, or inadvertent embolization of hemostatic agents such as Gelfoam slurry [4,5]. In some cases, underlying hypercoagulable states or cirrhotic architecture may predispose patients to thrombus formation [6,7]. Although literature on postbiopsy PVT is sparse, prior isolated case reports have documented multidisciplinary management [8,9].

We report the case of a 51-year-old male with common variable immunodeficiency (CVID), chronic liver enzyme elevation, and imaging findings consistent with cirrhosis, who underwent ultrasound-guided random liver biopsy. Within minutes of the procedure, he developed acute abdominal pain and was found to have subsegmental portal vein thrombosis on contrast-enhanced CT. Of note, the final biopsy pathology revealed only mild steatosis and portal chronic inflammation without significant fibrosis, a discordance with the preoperative imaging impression that is discussed in detail below. This case highlights the importance of early recognition and intervention in managing rare vascular complications following liver biopsy in patients with multiple comorbidities.

## Case report

A 51-year-old male with a complex medical history including common variable immunodeficiency (CVID), hepatic steatosis, and prior total parenteral nutrition (TPN) use, presented for evaluation of chronic liver enzyme elevation and imaging findings concerning for cirrhosis. Abdominal CT and MRI revealed nodularity of the hepatic surface and cirrhotic morphology, prompting further assessment to determine the underlying etiology (Fig. 1). The differential diagnosis included metabolic dysfunction-associated steatohepatitis (MASH), CVID-related hepatopathy, and TPN-associated hepatic injury [[10], [11], [12]]. Based on recommendations from hepatology, a liver biopsy was performed to obtain tissue for diagnosis.

**Fig. 1:**
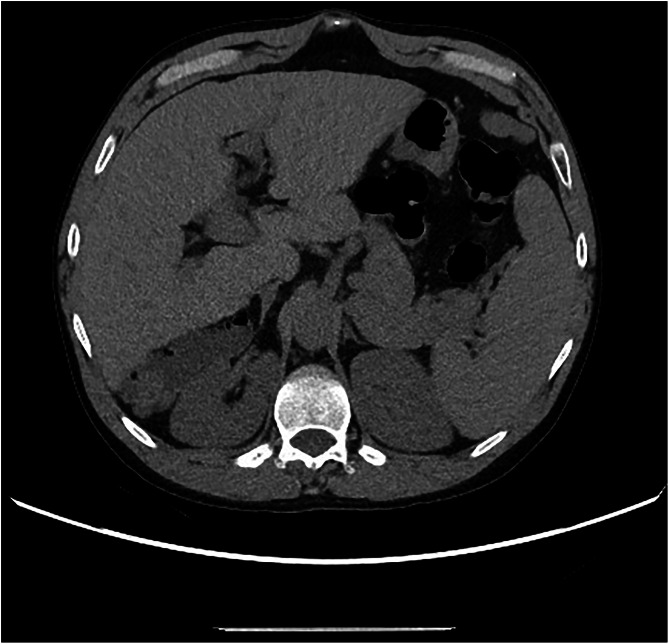
Prebiopsy contrast-enhanced CT. Axial contrast-enhanced CT obtained in the portal venous phase prior to liver biopsy demonstrating cirrhotic liver morphology with surface nodularity, irregularity of the hepatic contour, and heterogeneous parenchymal enhancement. The portal venous system is patent at baseline without evidence of thrombosis. These imaging features, while consistent with cirrhotic architecture, may reflect CVID-associated nodular regenerative hyperplasia (NRH) or advanced hepatic fibrosis rather than true histologic cirrhosis, as evidenced by the subsequent biopsy findings. Images serve as the baseline comparison for postprocedural findings.

An ultrasound-guided random liver biopsy targeting the left hepatic lobe was performed in the interventional radiology suite (Fig. 2). Moderate sedation was administered using fentanyl and midazolam, along with local lidocaine anesthesia. The procedure was technically successful, with placement of an 18-gauge coaxial biopsy needle and retrieval of 3 core samples. Minimal blood loss was observed (<15 mL), and no immediate procedural complications were noted. Postprocedural tract embolization was performed with Gelfoam slurry to minimize bleeding risk [13].

**Fig. 2:**
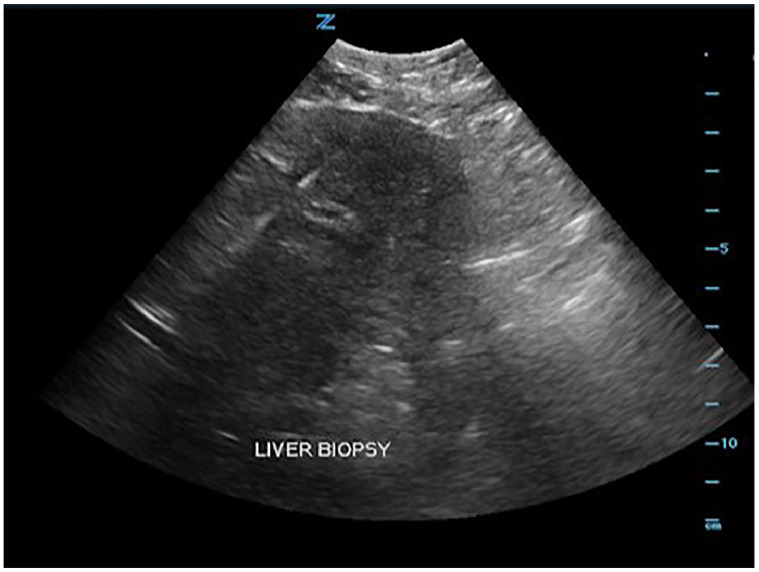
Liver biopsy ultrasound. Ultrasound-guided random liver biopsy targeting the peripheral segment of the left hepatic lobe using an 18-gauge coaxial CorVect needle. Three core samples were obtained under real-time sonographic guidance. The image captures needle placement before sampling.

However, within minutes of biopsy completion, the patient developed severe epigastric and right upper quadrant abdominal pain accompanied by chills and clinical signs of jaundice. A postprocedural CT scan was performed in the portal venous phase, demonstrating cirrhotic liver morphology with heterogeneous enhancement of the left hepatic lobe in the biopsy region. The observed heterogeneous left lobe enhancement was interpreted as a transient hepatic attenuation difference (THAD) reflecting compensatory arterial inflow in response to focal portal venous obstruction, rather than true parenchymal ischemia or infarction (Fig. 3). On portal venous phase imaging, a subsegmental portal venous filling defect in segment 3 was identified, consistent with acute thrombosis. No evidence of active bleeding or perihepatic hematoma was present. The patient was admitted for intravenous pain control and further evaluation.

**Fig. 3:**
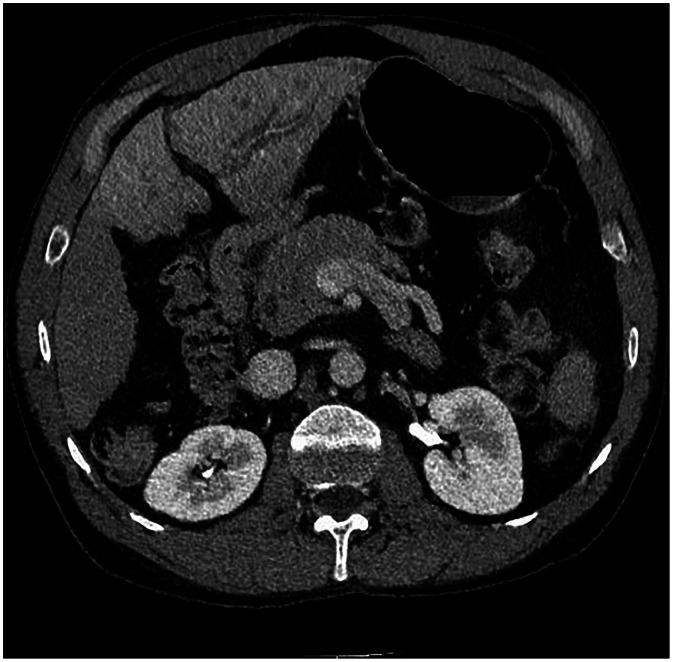
Postbiopsy CT–active portal vein thrombosis. Axial contrast-enhanced CT performed in the portal venous phase within hours of liver biopsy demonstrating heterogeneous enhancement of the left hepatic lobe in the biopsy region. This heterogeneous enhancement pattern is consistent with a transient hepatic attenuation difference (THAD), reflecting compensatory arterial inflow in response to focal portal venous obstruction, and should not be confused with parenchymal ischemia or infarction. Arrow indicates the subsegmental portal venous filling defect in segment 3, consistent with acute thrombosis. No perihepatic hematoma or active extravasation is identified.

During hospitalization, repeat CT imaging on postoperative day 1 showed persistent thrombosis of the segment 3 portal venous branch along with a new filling defect in the left main portal vein, consistent with proximal thrombus extension (Fig. 4). Imaging did not demonstrate embolic material within the main portal vein; thus, Gelfoam migration remains a hypothesized rather than confirmed contributing mechanism. Laboratory studies revealed marked transaminitis, with AST and ALT exceeding 900 U/L, hyperbilirubinemia peaking at 13.7 mg/dL, and thrombocytopenia (Table 1). No evidence of hemoperitoneum, abscess, or biliary obstruction was present on imaging. Magnetic resonance cholangiopancreatography (MRCP) showed no bile duct abnormalities or hemobilia (Fig. 5).

**Fig. 4:**
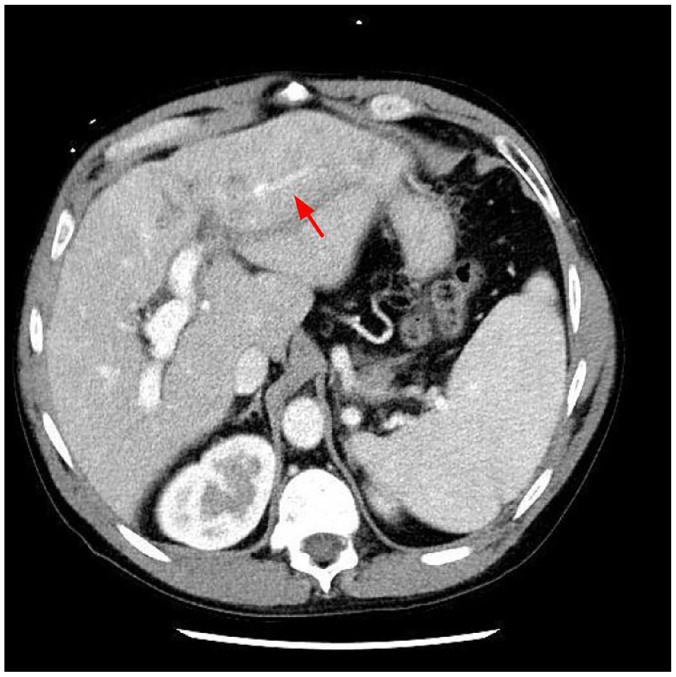
Follow-up CT–thrombus extension. Axial contrast-enhanced CT obtained in the portal venous phase on postprocedure day 1 demonstrating an irregular, attenuated subsegmental portal vein branch within hepatic segment 3. This is contributing to mild focal portal venous congestion in segment 3, consistent with proximal thrombus propagation. Arrow indicates the left main portal vein filling defect. No embolic material is identified in the main portal vein, no intestinal ischemia, infarction, or hemorrhagic complications are present.

**Table 1:** Summary of laboratory abnormalities (selected).

Date	AST (U/L)	ALT (U/L)	Total bili (Mg/dL)	Platelets (K)	INR
Preop	61	66	0.5	179	0.9
Postop 0	452	224	6.0	124	0.9
Postop 1	967	736	13.7	107	0.9
Postop 2	508	543	3.1	81	1.1

Sequential laboratory values demonstrating progressive transaminase and bilirubin elevation following ultrasound-guided liver biopsy, peaking on postprocedure day 1 (AST 967 U/L, ALT 736 U/L, total bilirubin 13.7 mg/dL), with subsequent improvement following initiation of anticoagulation therapy. Thrombocytopenia developed concurrently with the acute thrombotic event. INR remained within normal limits throughout the admission, consistent with a provoked rather than coagulopathy-driven thrombosis. Postop 0 values reflect same-day index procedure day laboratory results obtained after biopsy completion.

AST, aspartate aminotransferase; ALT, alanine aminotransferase; Bili, bilirubin; INR, international normalized ratio.

**Fig. 5:**
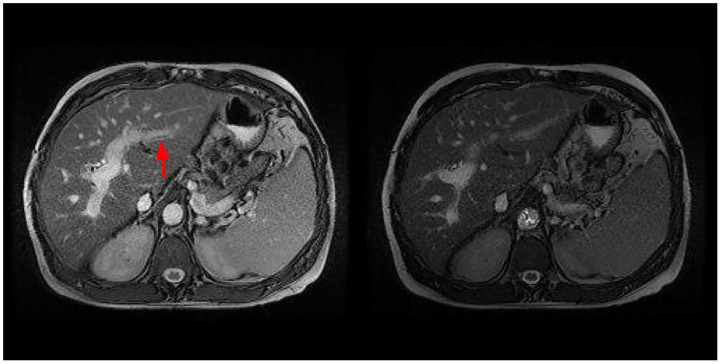
MRCP–exclusion of biliary complications. Magnetic resonance cholangiopancreatography (MRCP) demonstrating a more uniform enhancement of hepatic segment 3 on early portal and portal-venous/equilibrium phase axial fat-suppressed T1-erighted images. The previously questioned irregular, attenuated segment 3 subsegmental portal vein branch now appears of near-normal caliber and enhancement, without a definite intraluminal filling defect or segmental perfusion abnormality. These findings are compatible with interval improvement/resolution of prior suspected focal portal venous compromise following conservative management with anticoagulation. There is little to no evidence of hemobilia, ductal dilatation, stricture, or obstruction. Findings exclude biliary etiology for the patient's jaundice and hyperbilirubinemia, which are attributed to hepatocellular dysfunction secondary to portal vein thrombosis and associated hepatic parenchymal injury.

Anticoagulation was initiated with an intravenous heparin drip, later transitioned to oral apixaban upon stabilization [14,15]. Consultations with interventional radiology, hepatology, and urology were obtained, with hematuria deemed likely related to anticoagulant therapy. The patient underwent serial laboratory monitoring and repeat imaging to evaluate disease progression and therapeutic response (Table 2).

**Table 2:** Imaging findings timeline.

Imaging date	Modality	Key findings
Postop 0	CT with contrast	Cirrhotic morphology; subsegmental segment 3 PVT; heterogeneous left lobe enhancement in biopsy region; delayed venous phase filling; no active bleeding or hematoma
Postop 1	CT with contrast	Persistent segment 3 PVT; new filling defect in the left main portal vein suggestive of thrombus extension; no hemoperitoneum or abscess
Postop 1	MRCP	Normal biliary tree; no hemobilia, ductal dilatation, or biliary obstruction

Sequential cross-sectional imaging demonstrating evolution of portal vein thrombosis from isolated subsegmental segment 3 involvement on portal venous phase CT (PostOp Day 0) to proximal extension into the left main portal vein (PostOp Day 1), with concurrent exclusion of hemorrhagic and biliary complications. The heterogeneous left lobe enhancement on PostOp Day 0 CT is consistent with a transient hepatic attenuation difference (THAD) rather than parenchymal ischemia. No embolic material was identified in the main portal vein on either CT acquisition.

MRCP, magnetic resonance cholangiopancreatography; PVT, portal vein thrombosis; THAD, transient hepatic attenuation difference.

The patient's liver function tests and bilirubin levels improved on postoperative day 2 with anticoagulation, and his abdominal pain gradually resolved. No signs of intestinal ischemia or worsening thrombosis were noted on follow-up imaging during the index hospitalization. He was discharged home on apixaban with plans for at least 2 months of therapy. Outpatient follow-up was arranged with hepatology, urology, and immunology services. On review of the liver biopsy pathology, hepatic parenchyma exhibited mild steatosis, portal chronic inflammation with ductular proliferation, and absence of significant fibrosis, a finding discordant with the preoperative imaging impression of cirrhotic morphology, as discussed below. No further interventional procedures were required, and the patient's status returned to baseline without recurrence of hematuria. No dedicated postdischarge cross-sectional imaging was performed at our home institution to assess portal vein recanalization status.

## Discussion

Portal vein thrombosis (PVT) is an uncommon but clinically significant complication following percutaneous liver biopsy. Although traditionally associated with cirrhosis, malignancy, or hypercoagulable states, PVT may also result from local endothelial injury caused by needle passage during biopsy in the presence of other comorbidities [16,17]. In this case, the patient's abrupt onset of abdominal pain and substantial laboratory abnormalities led to immediate vascular imaging, which confirmed subsegmental PVT. The absence of a perihepatic hematoma and the use of Gelfoam slurry for tract embolization suggested a thrombotic rather than hemorrhagic mechanism. Studies have suggested that cirrhotic architecture and impaired portal flow can contribute to vascular stasis and increased thrombotic risk in this population [6,18]. Additionally, inadvertent migration of embolic agents such as Gelfoam into portal venous branches has been implicated as a potential thrombogenic mechanism in prior case reports [5,8]. However, as direct imaging confirmation of embolic material within the portal system was not obtained in this case, Gelfoam migration should be characterized as a plausible hypothesis rather than a confirmed etiology.

The pathophysiology of postbiopsy PVT in this patient can be conceptualized through the framework of Virchow's triad: endothelial injury, blood flow stasis, and a hypercoagulable state. First, direct mechanical trauma from needle passage caused focal endothelial disruption of a subsegmental portal venous branch. Second, the underlying hepatic architecture, whether representing true cirrhosis or advanced fibrosis, likely resulted in reduced and turbulent portal venous flow, contributing to local stasis. Third, although the patient's INR was normal, the systemic inflammatory state associated with CVID and the recent procedure may have contributed to a relative prothrombotic state. In cirrhotic patients, PVT has been attributed to all 3 vertices of Virchow's triad, with reduced portal flow velocity representing a particularly significant contributor [19].

An important finding in this case was the discordance between the preoperative imaging appearance of cirrhotic morphology and the biopsy-proven histology, which revealed only mild steatosis and portal chronic inflammation without significant fibrosis. This discordance is a well-recognized limitation of both percutaneous liver biopsy and cross-sectional imaging. Liver biopsy is subject to sampling error, as a needle core represents less than 1/50,000th of the total liver volume, and histologic staging can vary significantly depending on the location sampled [20]. Conversely, imaging features such as surface nodularity and parenchymal heterogeneity may reflect noncirrhotic portal hypertension or nodular regenerative hyperplasia (NRH)—conditions well-documented in CVID-related hepatopathy—rather than true histologic cirrhosis [[21], [22], [23]]. The imaging findings in this patient are most consistent with CVID-associated NRH or advanced fibrotic architecture. Clinicians should be aware that imaging overcall of cirrhosis is possible in this population, and that the absence of significant histologic fibrosis does not negate the elevated PVT risk conferred by portal hypertension and vascular architecture changes in CVID-related liver disease.

Several predisposing factors likely converged in this case, including the abnormal hepatic vascular architecture associated with CVID-related liver disease and the procedural insult itself. CVID-related liver disease occurs in approximately 10% of affected patients and is characterized by cholestatic liver enzyme patterns, nodular regenerative hyperplasia (NRH), and noncirrhotic portal hypertension [[21], [22], [23]]. In CVID patients, liver involvement is associated with immune dysregulation, reduced survival, longer hospitalizations, and higher mortality rates compared to CVID patients without hepatic complications [24,25]. Despite a normal INR and no baseline antithrombotic therapy, these conditions may have created a vulnerable vascular microenvironment predisposing to thrombosis following mechanical injury.

A hypercoagulable evaluation was not performed during the acute admission, as the patient's INR was normal and the clinical context was consistent with a provoked thrombotic event. While routine testing for inherited thrombophilia is not universally recommended in provoked PVT, testing for antiphospholipid antibodies may be clinically relevant given that antiphospholipid syndrome preferentially warrants vitamin K antagonist therapy rather than direct oral anticoagulants [26]. The absence of a formal hypercoagulable workup is acknowledged as a limitation of this report.

The patient's postoperative management included early contrast-enhanced CT and MRCP to exclude biliary or hemorrhagic complications, followed by prompt initiation of anticoagulation with intravenous heparin and transition to apixaban after stabilization. Multidisciplinary coordination with hepatology, urology, and interventional radiology was essential, particularly in managing anticoagulation-related hematuria. Published data support individualized risk stratification in postbiopsy PVT, with favorable outcomes when anticoagulation is initiated promptly in stable patients [27,28]. Recanalization occurs in 42%-44% of acute cases with early anticoagulation, and partial recanalization is achieved in over half of patients [14,15,29]. Regarding the choice of anticoagulant, direct oral anticoagulants (DOACs) such as apixaban have increasingly been studied in cirrhotic and portal hypertensive patients and represent an emerging alternative to traditional anticoagulation, with comparable safety profiles reported in recent systematic reviews [30]. Long-term anticoagulation is typically reserved for patients with recurrent thrombosis or underlying prothrombotic conditions [31]. In this case, the patient's liver function improved steadily, and no further interventional procedures were required.

This case underscores the importance of maintaining high clinical suspicion for vascular complications following liver biopsy, especially in patients with chronic liver disease and systemic immune dysregulation. Rapid diagnosis, imaging, and therapeutic intervention led to clinical recovery without lasting sequelae. Limitations of this report include the absence of postdischarge follow-up imaging to assess portal vein recanalization status and the lack of a formal hypercoagulable evaluation during the index admission. This report adds to the limited literature on postbiopsy PVT and highlights the convergence of multiple risk factors in its pathogenesis including abnormal hepatic vascular architecture, CVID-related immune dysregulation, procedural endothelial injury, and potential hemostatic agent migration. Clinicians performing liver biopsies in patients with complex medical histories should remain alert for early signs of thrombotic complications and initiate prompt diagnostic workup and anticoagulation when clinically indicated.

## Patient consent

Written informed consent was obtained from the patient for publication of this case report.
